# Distinct COPD subtypes in former smokers revealed by gene network perturbation analysis

**DOI:** 10.1186/s12931-023-02316-6

**Published:** 2023-01-25

**Authors:** Kristina L. Buschur, Craig Riley, Aabida Saferali, Peter Castaldi, Grace Zhang, Francois Aguet, Kristin G. Ardlie, Peter Durda, W. Craig Johnson, Silva Kasela, Yongmei Liu, Ani Manichaikul, Stephen S. Rich, Jerome I. Rotter, Josh Smith, Kent D. Taylor, Russell P. Tracy, Tuuli Lappalainen, R. Graham Barr, Frank Sciurba, Craig P. Hersh, Panayiotis V. Benos

**Affiliations:** 1 Department of Computational and Systems Biology, University of Pittsburgh School of Medicine, Pittsburgh, PA USA; 2 Joint CMU-Pitt PhD Program in Computational Biology, Pittsburgh, PA USA; 3 Division of General Medicine, Columbia University Medical Center, New York, NY USA; 4 New York Genome Center, New York, NY USA; 5 Division of Pulmonary Medicine, Department of Medicine, University of Pittsburgh, Pittsburgh, PA USA; 6 Channing Division of Network Medicine, Brigham and Women’s Hospital, Boston, MA USA; 7 The Broad Institute of MIT and Harvard, Cambridge, MA USA; 8 Department of Pathology and Laboratory Medicine, Larner College of Medicine, University of Vermont, Burlington, VT USA; 9 Department of Biostatistics, University of Washington, Seattle, WA USA; 10 Department of Systems Biology, Columbia University, New York, NY USA; 11 Department of Medicine, Division of Cardiology, Duke Molecular Physiology Institute, Duke University Medical Center, Durham, NC USA; 12 Center for Public Health Genomics, University of Virginia, Charlottesville, VA USA; 13 The Institute for Translational Genomics and Population Sciences, Department of Pediatrics, The Lundquist Institute for Biomedical Innovation at Harbor-UCLA Medical Center, Torrance, CA USA; 14 Northwest Genome Center, University of Washington, Seattle, WA USA; 15 Department of Biochemistry, Larner College of Medicine, University of Vermont, Burlington, VT USA; 16 Science for Life Laboratory, Department of Gene Technology, KTH Royal Institute of Technology, Stockholm, Sweden; 17 Department of Epidemiology, University of Florida, 2004 Mowry Rd, Gainesville, FL 32603 USA

**Keywords:** COPD, Graphical models, Gene expression, Disease subtypes

## Abstract

**Background:**

Chronic obstructive pulmonary disease (COPD) varies significantly in symptomatic and physiologic presentation. Identifying disease subtypes from molecular data, collected from easily accessible blood samples, can help stratify patients and guide disease management and treatment.

**Methods:**

Blood gene expression measured by RNA-sequencing in the COPDGene Study was analyzed using a network perturbation analysis method. Each COPD sample was compared against a learned reference gene network to determine the part that is deregulated. Gene deregulation values were used to cluster the disease samples.

**Results:**

The discovery set included 617 former smokers from COPDGene. Four distinct gene network subtypes are identified with significant differences in symptoms, exercise capacity and mortality. These clusters do not necessarily correspond with the levels of lung function impairment and are independently validated in two external cohorts: 769 former smokers from COPDGene and 431 former smokers in the Multi-Ethnic Study of Atherosclerosis (MESA). Additionally, we identify several genes that are significantly deregulated across these subtypes, including *DSP* and *GSTM1*, which have been previously associated with COPD through genome-wide association study (GWAS).

**Conclusions:**

The identified subtypes differ in mortality and in their clinical and functional characteristics, underlining the need for multi-dimensional assessment potentially supplemented by selected markers of gene expression. The subtypes were consistent across cohorts and could be used for new patient stratification and disease prognosis.

**Supplementary Information:**

The online version contains supplementary material available at 10.1186/s12931-023-02316-6.

## Introduction

Chronic obstructive pulmonary disease (COPD) is a heterogeneous disease, including emphysema and small and large airways disease [1, 2]. COPD diagnosis [3] is based on spirometric measures reflecting reduced airflow obstruction, specifically a reduced ratio of forced expiratory volume in 1-s (FEV_1_) to forced vital capacity (FVC) less than 0.70 [4]. But this definition does not account for the vast heterogeneity observed in COPD cases in terms of the rate of progression of the disease [5], response to treatment [6–8], symptom burden [9], inflammatory response [10], and lung physiology [11]. Therefore, there has been tremendous interest in identifying COPD subtypes that reflect differences in these disease aspects [12, 13]. Well-characterized subtypes with readily assessable biomarkers would allow for the selection of high-risk COPD populations for therapeutic intervention and patient stratification leading to more highly-powered clinical trials. Molecular subtyping could also help to identify rare genetic variants and individuals at elevated risk for development of the disease [14].

Disease subtyping has been relatively successful in asthma [15], but efforts in COPD have proven more difficult. Previous attempts to subtype COPD have been limited due to lack of reproducibility and constraints in study design. Another limitation to COPD subtyping efforts is the barrier to validating and interpreting subtypes that are based on clinical characteristics (e.g., spirometry, body mass index). Some studies have tried to circumvent this problem by withholding a pre-defined subset of clinical characteristics at the clustering step and then using those to assess the resulting clusters [16]; however, this raises the question of whether the holdout set is representative of the population. While it is possible to find distinct groups of subjects regarding these clinical variables, these classifications are unlikely to identify novel disease mechanisms.

Incorporation of genomic information can greatly enhance the relevance of COPD subtypes. Peripheral blood gene expression is an attractive candidate for potential biomarkers because it is easily accessible. One previous study identified four COPD clusters based on blood gene expression with a non-negative matrix factorization approach [17]. These clusters of subjects promisingly varied in the severity of their disease, but, because the study relied on microarray gene expression data, discovery was limited to the genes included on those platforms.

We recently developed a new method for evaluating gene network perturbations in single samples (*single sample Network Perturbation Assessment*, ssNPA) [18]. ssNPA uses probabilistic graphs [19–22] to estimate the gene network from a set of reference (control) samples and assesses perturbations in each individual disease sample. ssNPA outperformed existing algorithms in identifying subgroups of samples based on these gene expression perturbation features and had superior clustering performance compared to gene expression itself [18] and other methods [23, 24]. In this paper, we apply ssNPA to the Genetic Epidemiology of COPD Study (COPDGene) and the Multi-ethnic Study of Atherosclerosis (MESA) data in order to identify and validate new COPD phenotypes solely from gene expression measured in peripheral blood samples.

## Methods

### COPD subtyping: discovery and validation cohorts

The COPDGene Study is a longitudinal study that aims to investigate the genetic basis of COPD susceptibility and progression. Our subtype discovery dataset consisted of 1211 COPDGene subjects for whom whole blood RNA-seq data were collected at the 5 year follow-up visit [25]. The first validation dataset included 1444 COPDGene participants that were sequenced later. These samples were not included in the training dataset and were processed independently. The second validation dataset consisted of 821 unrelated MESA participants. MESA is an ongoing prospective cohort study that recruited over 6000 participants in six communities throughout the United States between 2000 and 2002 [26]. Peripheral blood mononuclear cell (PBMC) gene expression was measured by RNA-seq at Exam 5 between 2010 and 2012. Detailed phenotype data (including spirometry and CT scan) were also collected at this exam.

### Reference subject selection

**Fig. 1:**
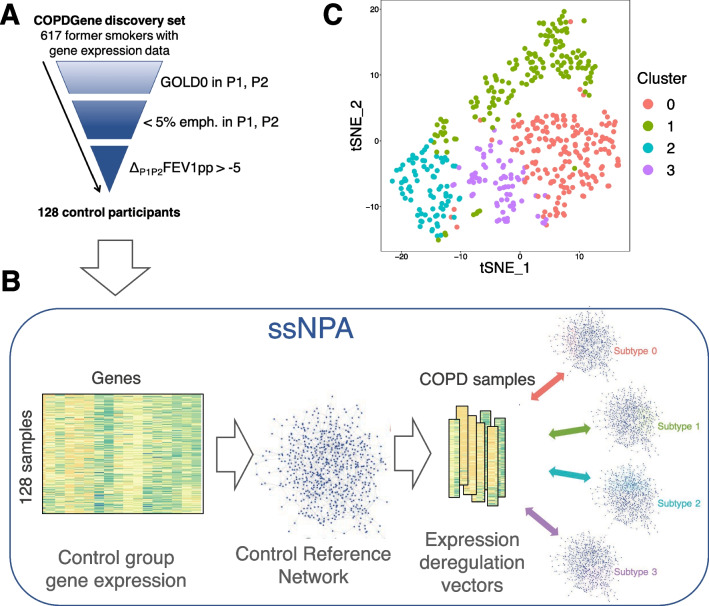
Overview of the subtyping procedure. **A** Selection of reference (control) samples from the COPDGene discovery cohort (617 former smokers). **B** Network deregulation procedure to identify COPD subtypes. **C** t-SNE plot of the four COPD sample clusters identified by ssNPA. Clusters 0 and 1 have similar clinical characteristics, as do clusters 2 and 3

The COPDGene RNA-seq samples were preprocessed as in the Additional file 1: Methods. The reference gene network was built on a group of former smokers, selected conservatively based on the following criteria (Fig. 1A): participant had (a) both Phase 1 (baseline) and Phase 2 (5-yr) visits; (b) normal spirometry both visits; (c) less than 5% percent emphysema in both visits (LAA-950); (d) less than 5% decrease in percent predicted FEV_1_ between the two visits. This filtering resulted in 128 reference samples (training dataset). To increase power, all remaining samples from participants who formerly smoked cigarettes and who did not meet the criteria for the reference group were included in the disease group, leaving 489 samples for subtype discovery.

For the COPDGene validation dataset, the same filtering criteria identified 149 control and 614 COPD former smoker samples. The MESA reference group was selected with similar criteria, except the threshold for FEV_1_ decline between Exam 3/4 and Exam 5 (< 3% predicted). Participants with no spirometry data were included in the non-reference group. This resulted in 104 reference and 327 case MESA samples. Table 1 summarizes the characteristics of these three study groups.

**Table 1:** Characteristics of the three study groups

	COPDGene discovery group		COPDGene validation group		MESA validation group	
	Reference group	Case group	Reference group	Case group	Reference group	Case group
Participants, *n*	128	489	149	614	104	327
Age, yr, mean (SD)	66.7 (8.8)	70.1 (7.7)	67.7 (8.6)	69.5 (7.6)	67.5 (8.3)	70.1 (9.3)
Sex, F, *n* (%)	78 (60.9)	216 (44.1)	91 (61.1)	290 (47.2)	53 (51.0)	137 (41.9)
Race, Non-Hispanic White, *n* (%)	111 (86.7)	457 (93.5)	138 (92.6)	562 (91.5)	45 (43.3)	177 (54.1)
Race, African American, *n* (%)	17 (13.3)	32 (6.5)	11 (7.4)	52 (8.5)	18 (17.3)	68 (20.8)
Race, Hispanic, *n* (%)	0 (0)	0 (0)	0 (0)	0 (0)	41 (39.4)	82 (25.1)
GOLD stage 0, *n* (%)	128 (100)	145 (29.7)	149 (100)	210 (34.2)	104 (100)	109 (33.3)
PRISm, *n* (%)	NA	62 (12.7)	NA	84 (13.7)	NA	0
GOLD stage 1, *n* (%)	NA	57 (11.7)	NA	74 (12.1)	NA	98 (30.0)
GOLD stage 2, *n* (%)	NA	130 (26.6)	NA	137 (22.3)	NA	0
GOLD stage 3, *n* (%)	NA	76 (15.6)	NA	85 (13.9)	NA	0
GOLD stage 4, *n* (%)	NA	18 (3.7)	NA	21 (3.4)	NA	0
Smoking history, pack-year, mean (SD)	37.2 (21.9)	45.9 (25.6)	35.5 (21.0)	43.6 (23.4)	12.7 (14.1)	16.8 (20.2)

NB: There were 120 participants in the MESA group for whom spirometry data was not available. These were all included in the case group, but GOLD stage could not be calculated

*GOLD *Global Initiative for Chronic Obstructive Lung Disease, *MESA* Multi-Ethnic Study of Atherosclerosis, *PRISm* Preserved Ratio Impaired Spirometry

### COPD subtyping from blood RNA-seq data

We used ssNPA, a network-based disease subtyping method, to learn COPD subtypes in the discovery cohort (Fig. 1B). The details of the process are presented in Additional file 1: Methods. To investigate the clinically relevant differences among the clusters of COPD samples we identified, we compared the values of 105 clinical variables across the clusters (Additional file 2: Table S1). These included spirometry, chest CT scan, symptom questionnaires, and white blood cell differentials as well as medical history, medication, and comorbidity information. The *p*-values were calculated using Kruskal–Wallis (continuous, ordinal) and Chi-squared test (binary). A false discovery rate (FDR) threshold of *q*-value < 0.05 was used for multiple test correction. Pairwise comparisons between cluster means were assessed by Wilcoxon test. Survival analysis was performed by the Kaplan–Meier method with the survfit function from the survival R package (v. 3.1-8).

To better understand how the clusters were separated, we considered the magnitude of the PCA loading for each feature. Gene features with the highest loading values in the top principal components correspond to the genes whose deregulation relative to the controls contributes the most to separating the clusters.

Finally, MESA and COPDGene validation cohort samples were assigned to one of the four subtypes using the following procedure described in Additional file 1: Methods.

## Results

### COPD clusters exhibit different clinical phenotypes

ssNPA separated the 489 COPD PBMC samples (all former smokers) into four clusters (Fig. 1C, Additional file 1: Fig. S8). The first two clusters were of roughly equal size (cluster 0 and cluster 1 have 37.0% and 31.9% of the samples, respectively). Cluster 2 and cluster 3 were smaller (with 16.2% and 14.9% of the samples). Mean age was similar in all clusters. Cluster 2 had a higher proportion of women and its subjects had a higher neutrophil and lower eosinophil percent, which are indicative of inflammation.

Statistical analyses identified clinical characteristics that are significantly associated with these clusters (Table 2). Overall, cluster 2 represented the most impaired subjects, with lower 6-min walk distance, more symptoms (measured by COPD Assessment Test, CAT) and dyspnea (measured by the Modified Medical Research Council Dyspnea Scale, MMRC), and the worst disease-related quality of life on the St. George’s Respiratory Questionnaire (SGRQ). However, spirometry (FEV_1_ percent predicted and FEV_1_/FVC) was similar in clusters 2 and 3, but worse than cluster 0 and 1. Clusters 0 and 1 have the best spirometric lung function, but ~ 4 times more subjects in cluster 0 are using inhaled corticosteroids (5.08% vs 1.3%; Table 2). Regardless, the percentage of corticosteroid usage in subjects of cluster 2 and cluster 3 is even higher (11.54%, 13.7%), making this feature significantly different between clusters. Diffusing capacity of carbon monoxide (DLCO) percent predicted was highest in cluster 0 (76.7%), but similar in the other 3 clusters (70.8, 69.1, 70.5% predicted, respectively). Cluster 3 had the greatest percent emphysema (12.03%), followed by cluster 2 (10.84%), while cluster 0 and cluster 1 had the least emphysema (8.59%, 8.47%) (Additional file 2: Table S1). However, these differences are not significant at the 5% FDR level (q-value = 0.068). Similarly, observed differences in Pi10 and Perc15 are not significant (q-value = 0.072–0.076).

**Table 2:** Clinical characteristics of COPD participants vary across clusters. The variables are sorted by descending significance

Variable	q-value	Cluster 0	Cluster 1	Cluster 2	Cluster 3	All reference participants
Participants, *n*		181	156	79	73	128
Sex, F, *n* (%)		80 (44.2)	63 (40.4)	42 (53.2)	31 (42.5)	78 (60.9)
Age, yr, mean (SD)		69.5 (7.8)	70.8 (7.3)	70.3 (7.9)	70.0 (8.1)	66.7 (8.8)
Cell types						
Lymphocyte percentage, mean (SD)	4.30E−04	28.07 (7.91)	26.97 (7.3)	23.13 (11.24)	24.84 (8.07)	30.91 (8.58)
Neutrophil percentage, mean (SD)	5.09E−04	60.3 (8.42)	60.76 (7.95)	65.94 (12.95)	62.78 (8.59)	57.73 (9.56)
Lymphocytes, K/µL, mean (SD)	4.74E−03	1.95 (0.65)	1.91 (0.61)	1.7 (0.79)	1.78 (0.6)	2.03 (0.7)
Neutrophils, K/µL, mean (SD)	3.11E−03	4.32 (1.43)	4.4 (1.41)	5.36 (2.23)	4.7 (1.69)	3.93 (1.47)
Medications						
Oral corticosteroids, *n* (%)	4.30E−04	3 (1.67)	1 (0.65)	8 (10.3)	0 (0)	1 (0.78)
Inhaled corticosteroids, *n *(%), mean (SD)	3.03E−03	9 (5.08)	2 (1.30)	9 (11.54)	10 (13.70)	3 (2.34)
Symptoms						
Distance walked, mean (SD)	2.73E−03	1384.88 (414)	1321.76 (403.63)	1142.14 (428.41)	1238.29 (407.99)	1520.71 (358.38)
Change in distance walked, mean (SD)	3.03E−03	− 53.9 (276.36)	− 134.48 (289.36)	− 208.35 (321.96)	− 172.2 (299.63)	− 87.64 (323.56)
SGRQ score: active, mean (SD)	3.11E−03	32.23 (27.42)	34.13 (29.9)	48.42 (31.68)	36.66 (25.38)	16.93 (20.25)
SGRQ score: total, mean (SD)	1.27E−02	19.99 (18.17)	21.87 (19.37)	30.47 (22.96)	22.04 (17.54)	9.24 (12.51)
MMRC dyspnea score, mean (SD)	1.27E−02	1.05 (1.29)	1.14 (1.28)	1.7 (1.6)	1.4 (1.29)	0.40 (0.86)
CAT score, mean (SD)	0.017	9.65 (6.95)	10.69 (7.47)	13.43 (8.84)	9.56 (6.82)	6.61 (5.73)
Spirometry						
FRC/TLC ratio, Thirona, mean (SD)	1.25E−03	0.56 (0.11)	0.6 (0.11)	0.63 (0.12)	0.61 (0.12)	0.51 (0.08)
FEV1, pre-BD, mean (SD)	0.018	1.95 (0.85)	1.88 (0.75)	1.62 (0.86)	1.77 (0.89)	2.53 (0.61)
FEV1/FVC ratio, mean (SD)	0.020	0.64 (0.15)	0.63 (0.16)	0.59 (0.17)	0.58 (0.16)	0.78 (0.06)
FEV1 percent predicted, mean (SD)	0.027	73.79 (24.65)	73.16 (24.49)	65.38 (26.96)	65.44 (27.12)	99.05 (11.33)
FEV1/FVC ratio, pre-BD, mean (SD)	0.034	0.62 (0.15)	0.62 (0.15)	0.58 (0.16)	0.58 (0.16)	0.75 (0.06)
DLCO percent predicted, adjusted, mean (SD)	0.042	76.69 (22.77)	70.84 (21.52)	69.09 (24.01)	70.52 (21.71)	92.08 (18.25)
BODE, mean (SD)	4.95E−03	1.11 (1.58)	1.26 (1.6)	1.92 (2.05)	1.82 (1.85)	0.22 (0.64)

P-values were calculated with a Kruskal-Wallis test for continuous and ordinal variables and or a Chi-squared test for discrete and binary variables and asses if there are differences in variable distribution among clusters. Variable means (standard deviations) are also reported for each COPD cluster and all control subjects for comparison. Variables of interest are included with a < 5% FDR cut-off. A full table of variables tested is provided in Additional file 2: Table S1

*BD *bronchodilator, *BODE* body mass index, airflow obstruction, dyspnea, and exercise capacity, *CAT* COPD Assessment Test, *DLCO* diffusing capacity for carbon monoxide, *FEV1* forced expiratory volume in 1-s, *FRC *functional residual capacity, *FVC *forced vital capacity, *MMRC* Modified Medical Research Council Dyspnea Scale, *SGRQ *St. George’s Respiratory Questionnaire, *TLC* total lung capacity

**Fig. 2:**
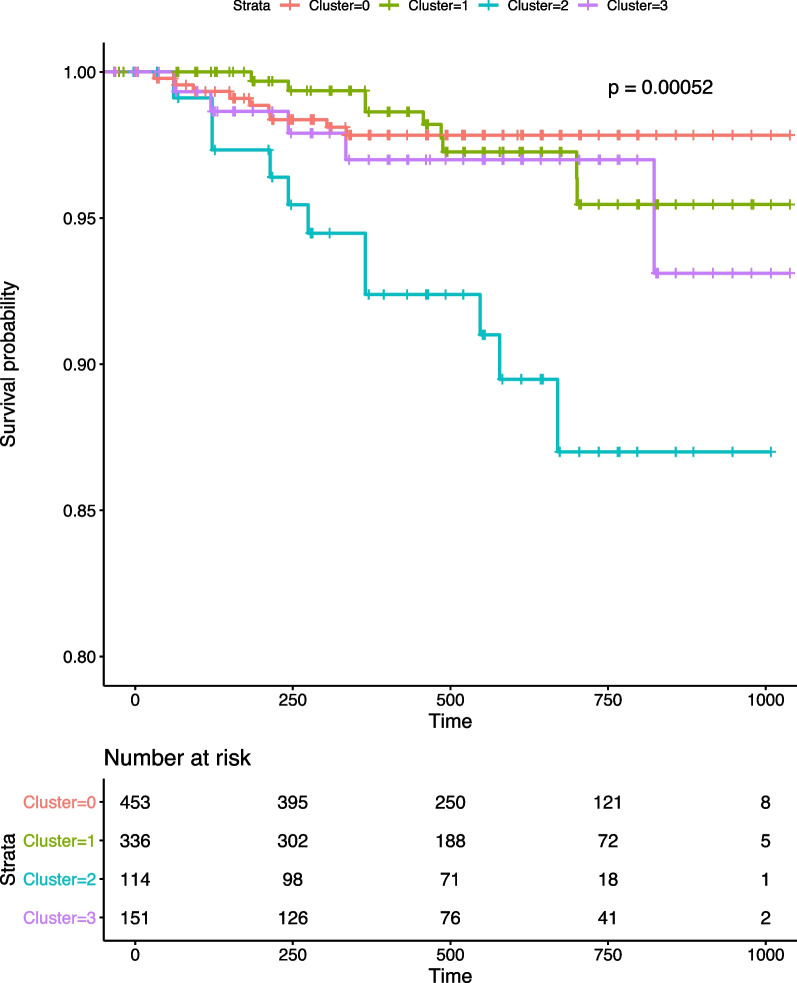
COPDGene discovery set participant survival varies by cluster. Survival analysis was performed with the Kaplan–Meier method, and the log-rank p-value from the score test is reported. Time measures days elapsed since Phase 2 visit

Survival analysis shows significant differences across the four clusters of COPDGene participants (Fig. 2; *p*-value < 0.001). This significance was driven by cluster 2, which has the worst outcome with the steepest decline in survival probability. Despite a lower FEV1 in cluster 3, survival probabilities in this cluster and cluster 1 began to decline similarly after approximately 2 years following study visit 2 to an intermediate level. Cluster 0 maintained the highest survival probability after this time. The observed survival differences between clusters 0, 1, and 3 are not significant. We also note that clustering based on spirometry characteristics alone (e.g., FEV1 percent predicted) did not produce differences in mortality (Additional file 1: Fig. S9).

We are aware that comorbidities may drive mortality either directly or through medications that people take because of them. Although our sample subtyping was based on blood gene expression and any comorbidities or medications that may influence our subtyping had to exert their effect through gene expression, we tested to see if our subtypes differ in terms of comorbidities. We found that none of the comorbidities recorded in COPDGene was significantly different between clusters (Additional file 1: Table S3.)

We further look in the variables that significantly differ between cluster 0 and cluster 1 (the two clusters with the highest FEV_1_). Both DLCO and the change in 6-min walk distance between the two visits are significantly worse in cluster 1 compared to cluster 0 (p-value = 0.011 and 0.017, respectively). DLCO differences are interesting given that percent emphysema was similar in the two clusters (Additional file 3: Table S2), which may indicate another process such as pulmonary vascular disease. A marker of pulmonary hyperinflation (FRC/TLC ratio) and a fatigue symptom also were found to be significantly different between these clusters (*p*-value = 0.005 and 0.036, respectively). However, the correlation between FRC/TLC ratio and adjusted DLCO percent predicted in each of these two clusters was quite low (− 0.41 and − 0.53 for cluster-0 and cluster-1, respectively), which is in line with previous studies [27, 28]. One explanation is that DLCO captures more than just emphysema, including pulmonary vascular disease and small airway disease [29].

### Subtype validation in independent datasets from COPDGene and MESA

**Fig. 3:**
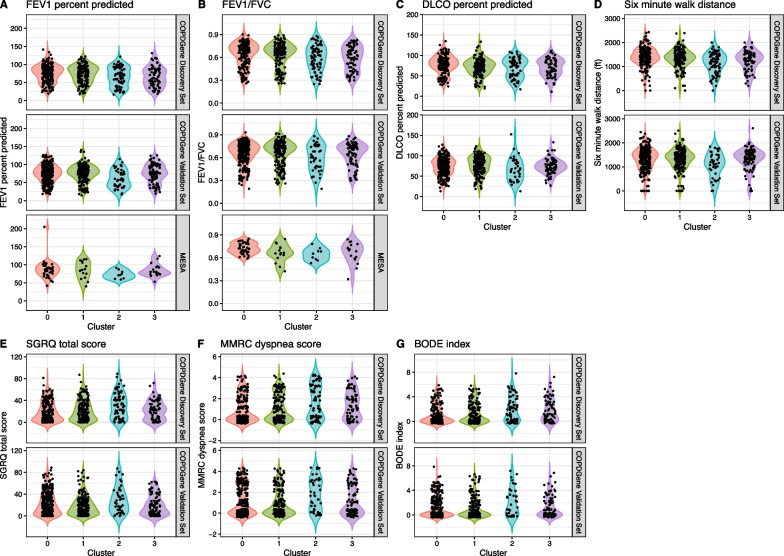
Clinical measures of disease severity and symptoms including (**A**) FEV1 percent predicted, (**B**) FEV1/FVC, (**C**) DLCO percent predicted (**D**) distance walked in six minutes, (**E**) SGRQ total score, (**F**) MMRC dyspnea score, and (**G**) BODE index, varying by cluster in the COPDGene discovery sample set as well as in the COPDGene and MESA validation sets. Some measures were not present in the MESA cohort. *BODE* body mass index, airflow obstruction, dyspnea, and exercise capacity, *FEV1* forced expiratory volume in 1 s, *FVC *forced vital capacity, *MMRC* Modified Medical Research Council Dyspnea Scale, *SGRQ* St. George’s Respiratory Questionnaire

We validated the ssNPA-generated subtypes by scoring new COPD samples from the validation groups using the model we learned from the COPDGene training cohort. We chose this type of validation because it demonstrates the ability to correctly assign new samples to these four subtypes, which is an attractive feature for future clinical application of these subtypes. Out of remaining 614 COPD samples in the COPDGene validation set, 288 were assigned to cluster 0, 193 to cluster 1, 42 to cluster 2, and 91 to cluster 3 (Additional file 1: Fig. S10). We emphasize that this cluster assignment was based solely on gene expression data. Therefore, we next checked to see if the clinical features that differed across clusters in the primary analysis followed the same trends across the clusters of these new samples. In general, we observed the same trends for the significant features when we looked at the discovery set of samples compared to the validation set of samples (Fig. 3), and many pairwise relationships between the cluster means of these features were replicated in the validation set (Additional file 1: Fig. S11). In general, we see higher concordance between the two cohorts when it comes to symptoms (CAT, MMRC Dyspnea and SGRQ Total scores) as well as forced expiratory flow (FEF 25–75).

The validation in the MESA cohort produced a similar result. 150 samples were assigned to cluster 0, 64 were assigned to cluster 1, 41 to cluster 2, and 72 to cluster 3 (Additional file 1: Fig. S12). Cluster 2 contained the most severe COPD phenotypes and contained the fewest MESA samples, and, conversely, cluster 0 exhibited the mildest disease and contains the most MESA samples. Again, we observed the same general trends for the clinical features in the MESA validation analysis as we did in the COPDGene discovery analysis (Fig. 3). Fewer of the pairwise comparisons of the means from the cluster of the clinical features were replicated in MESA, likely due to the smaller number of participants for whom these phenotypes were measured (Additional file 1: Fig. S13).

### ssNPA identifies a list of candidate genes deregulated in COPD

We further analyzed the identified COPD subtypes to investigate the molecular differences among them, which lead to different clinical phenotypes. We examined the gene deregulation features that had the largest PCA loadings to identify the genes that make the largest contribution to the clustering of the COPDGene discovery dataset (Additional file 1: Table S4). We focused on the top five loadings for each of the first six principal components (PCs) that were used to cluster the subjects and found that many of the genes came up more than once, including *DSP* and *GSTM1*, which have been previously associated with COPD. For many of these genes, we observe large differences across clusters in the distributions of deregulation magnitude (Additional file 1: Fig. S14), even between clusters whose subjects have similar lung function.

## Discussion

COPD subtyping is essential for not only understanding the diversity of molecular mechanisms of the disease, but also to aid in the development of new intervention strategies. Here we present a new clustering of COPD former smokers based on PBMC gene expression. The focus of this work was restricted to former smokers because we wanted to eliminate biases, since current smoking status has a large impact in gene expression. This clustering was the result of a novel network deregulation-based approach (ssNPA), which has been shown to outperform many standard methods in sample clustering [18]. We identify four COPD subtypes, which exhibit different degrees of symptom presentation, exercise capacity and mortality. Two of the clusters (cluster 0 and cluster 1) have similar (milder) impairment in spirometry, but show differences in DLCO, disease progression and mortality. The other two clusters (cluster 2 and cluster 3) have similar levels of lung function impairment, which is significantly worse than clusters 0 and 1. Compared to cluster 3, subjects in cluster 2 have more symptoms, lower 6-min walk distance, higher neutrophil count and worse survival despite similar reductions in FEV1 percent predicted and FEV1/FVC. Cluster 3 subjects have the most emphysema, although the differences are not significant.

We show that these clusters are stable by validating them using (1) additional COPDGene samples and (2) the MESA study cohort. To demonstrate the utility of our subtyping method for future patient classification, the samples from the two validation cohorts were assigned to one of these four clusters based on their own gene network deregulation vectors (instead of re-clustering these cohorts). We find that the clinical differences of the new sets of samples remained largely the same, which not only validates our findings but also demonstrates the ability of accurately assigning new samples to these four clusters. Unsurprisingly, the distribution of subtypes in MESA is skewed to include more in cluster 0 (mildest disease phenotype), since MESA enrolled subjects representing the general population. By contrast, COPDGene is a case–control study of COPD, so this distribution of MESA samples is consistent with our expectations.

Previous results in COPD subtype identification have proven difficult to replicate. For example, the number of identified subtypes generally varies from 2 to 5, and women and participants with mild disease are generally underrepresented [30]. One study applied a consistent clustering analysis to 10 independent cohorts and found only modest reproducibility across cohorts, but had more success with a continuous PCA-based projection of the individuals [31]. The authors suggest that the disease is best represented as a COPD continuum instead of separate and mutually exclusive subtypes. However, this interpretation does not account for the suspected varied genetic basis of COPD and, without clear cut-off points along the continuum, the practical utility is limited. Another study also applied a network-based clustering approach to blood microarray data and identified four clusters [17]. These clusters differed in spirometry and emphysema, but the network component in that study was coming from existing knowledge (STRING database), which has its own biases and limitations.

Next, we investigate the underlying molecular changes and how they may be implicated in the mechanism of the disease. Several of the genes whose deregulation drive the clustering to subtypes have previously been noted as having a role in COPD. Desmoplakin (*DSP*, 6p24.3) was identified in a genome-wide association study (GWAS) of COPD as one of 22 genes containing a top coding variant (rs2076295) [32]. *DSP* is a desmosomal protein that plays an essential role in cell–cell linkages, especially in epidermis and cardiac muscle [33, 34]. DSP variants have also been associated with idiopathic pulmonary fibrosis [35], although these variants may be protective against COPD [32]. This GWAS was included 15,256 COPD cases and 47,936 controls. This locus also colocalized with an expression quantitative trait locus (eQTL) from another lung tissue dataset that included subjects with COPD [36]. In another study, the locus was associated with change two quantitative measures of emphysema, percentage of low-attenuation area less than -950 Hounsfield units (%LAA-950) and adjusted lung density [37]. Recently, the variant was shown to regulate DSP expression in airway epithelial cells, and loss of DSP expression led to increased expression of extracellular matrix-related genes and cell migration [38].

Another gene we identified, *GSTM1* (gluthathione S-transferase μ 1, 1p13.3), belongs to a family of enzymes that are relevant for lung disease, likely through their roles in detoxifying electrophilic compounds, such as cigarette smoke and environmental toxins [39]. A homozygous *GSTM1*-null genotype has been associated with lung cancer pathogenesis [40, 41], emphysema [42, 43], and COPD susceptibility [44, 45]. However, *GSTM1* has not been previously identified by COPD GWAS, although the presumed functional variation is a gene deletion and not a single nucleotide polymorphism that would be included in GWAS chips.

Even though cluster 0 and cluster 1 had similar lung function, we identify a number of genes whose deregulation is different between these clusters. For example, the deregulation of *CTNNA2* and *SLC44A5* is higher in cluster 0 compared to cluster 1, and the deregulation of *MRGPRE* was lower in cluster 0 compared to cluster 1. Similarly, in cluster 2 and cluster 3 (also similar lung function), the deregulation of several genes, including *MUC16*, *ZMAT4*, *GSTM1*, *MRGPRE*, and *ADAM29*, differed between these two clusters. These observations indicate the presence of different underlying molecular mechanisms despite similar lung function.

The list of genes we have identified provides important insights into the molecular mechanism of susceptibility, such as the role of environmental toxin processing, and progression, including pathways involved in extracellular matrix organization. Several of the genes on the list such as Fibroblast Growth Factor 9 (*FGF9*) have not been specifically cited for an association with COPD, but they code for important signaling proteins and may play a role in lung development or airway remodeling.

As this is study is not meant to investigate the detailed molecular mechanisms of the four subtypes, we mention these genes as a proof-of-principle of our method. Future studies could investigate the role of the molecular mechanisms based on our results.

## Conclusions

Using the ssNPA method on blood gene expression data, we identify and validate four clusters of former smokers with COPD, which correspond to clinically relevant disease subtypes, reflecting differences in severity, symptoms and mortality. These differences are not fully reflected by lung function impairment alone. Furthermore, the focus on differential regulation at the gene level provides insight into the disease mechanisms that differentiate COPD cases from the control group of subjects without COPD. We identify a set of genes whose deregulation drives the subtype separation. Several of these genes have previously described connections to COPD, although some new genes emerged as well. The network learning and gene selection were completely unbiased, using no prior knowledge of clinical characteristics, disease mechanism or biology pathways. Finally, we show that ssNPA is a flexible general framework for disease subtyping. As more omics data become available through COPDGene and other studies, future work could incorporate genetic variant, epigenetic, proteomic, or metabolomic variables into the network learning and feature calculations that would provide a multi-layered, more complete picture of the molecular pathology and heterogeneity of COPD.

## Supplementary Information

**Additional file 1: Figure S1.** PCA of COPDGene primary analysis RNA-seq data colored according to batch (A) before and (B) after batch correction. Batch detection with guided principal component analysis showed strong batch effects before batch correction (p < 0.001) that were removed after batch correction (p = 0.538). **Figure S2.** PCA of COPDGene validation RNA-seq dataset colored according to batch (A) before and (B) after batch correction. Batch detection with guided principal component analysis showed strong batch effects before batch correction (p = 0.001) that were removed after batch correction (p = 0.937). **Figure S3.** PCA of MESA validation RNA-seq dataset colored according to batch (A) before and (B) after batch correction. Batch detection with guided principal component analysis showed strong batch effects before batch correction (p = 0.003) that were removed after batch correction (p = 1). **Figure S4.** Clustering tree illustrates the stability of clusters over a range of values for clustering resolution (res). We chose 4 as optimal number of clusters, because cluster number and content (samples) remains constant for res=0.6 to 0.9. When res>0.9 produced some subclusters of these four, but samples did not move across the four branches extending from these clusters. **Figure S5.** PC elbow plot of the COPDGene discovery set ssNPA features. We heuristically chose 6 principal components for clustering because they captured a large percentage of the variance in the data. **Figure S6.** Clustering tree illustrates the stability of clusters over a range of values for k in the kNN classification for the COPDGene validation analysis. We chose k=3 because the clusters were stable by this value. **Figure S7.** Clustering tree illustrates the stability of clusters over a range of values for k in the kNN classification for the MESA validation analysis. We chose k=3 because the clusters were stable by this value. **Figure S8.** Participant GOLD stage composition according to cluster. The reference group was composed of only GOLD 0 participants by design. **Figure S9.** Clustering based on FEV1 percent predicted does not sufficiently separate COPD individuals with different mortalities. **Figure S10.** COPDGene validation samples were projected into the same PCA space as the discovery analysis and assigned to clusters with kNN. Density clouds show the distribution of samples in each cluster from the discovery analysis. Individual points represent validation COPDGene samples and are colored according to the cluster to which they were assigned. **Figure S11.** Heatmaps display the p-value bins for the inter-cohort pairwise comparisons of cluster means by Wilcoxon test for: **physiology** **(A)** FEV1 percent predicted, **(B)** FEV1/FVC, **(C)** DLCO, **(D)** FRC/DLC ratio, **(E)** distance walked in 6 minutes; **symptoms (F)** SGRQ total score, **(G)** MMRC dyspnea score, and **(H)** CAT score. The upper right triangle shows the pairwise comparisons between cluster in the COPDGene discovery set, and the lower left triangle shows the comparisons between clusters in the COPDGene validation set. Blue (red) arrows indicate concordance in the significance (non-significance) of the comparisons between the two cohorts. **Figure S12.** MESA validation samples were projected into the same PCA space as the discovery analysis and assigned to clusters with kNN. Density clouds show the distribution of samples in each cluster from the discovery analysis. Individual points represent validation MESA samples and are colored according to the cluster to which they were assigned. **Figure S13.** Heatmaps display the p-value bins for the pairwise comparisons of cluster means by Wilcoxon test for (A) FEV1 percent predicted, (B) FEV1/FVC, (C) FEF 25-75%, and (D) percent emphysema. The upper right triangle shows the pairwise comparisons between cluster in the COPDGene discovery set, and the lower left triangle shows the comparisons between clusters in the COPDGene validation set. Blue (red) arrows indicate concordance in the significance (non-significance) of the comparisons between the two cohorts. **Figure S14.** ssNPA feature values show a difference in the degree of deregulation of (A) MUC16, (B) ZMAT4, (C), GSTM1, (D) CTNNA2, (E) MRGPRE, (F) SLC44A5, (G) ADARB2, and (H) ADAM29 across clusters. Wilcoxon test p-values highlight where there are differences in the distributions between clusters 0 and 1 and between clusters 2 and 3. **Table S3. **Analysis of various COPDGene comorbidities did not show any significant difference between the four identified subtypes. All comorbidities recorded at the time blood samples were collected. *p*-val: chi-square *p*-value. **Table S4. **The genes with the top 5 loadings for each of the first 6 PCs used for clustering the COPD samples in the training COPDGene dataset. Genes are sorted by decreasing contribution to the clustering (sum of the absolute values of the loadings across the first 6 PCs). Loading value is not provided if gene did not rank among the top 5 loadings for a given PC. The sample clustering is driven by differences in the regulation of these genes.

**Additional file 2: Table S1.** Excel file containing this table is attached. Clinical characteristics of COPD participants vary across clusters. The variables are sorted by descending significance. P-values were calculated with a Kruskal-Wallis test for continuous and ordinal variables and or a Chi-squared test for discrete and binary variables and asses if there are differences in variable distribution among clusters. Variable means (standard deviations) are also reported for all COPD participants overall, each COPD cluster, and all control subjects for comparison.

**Additional file 3: Table S2.** Excel file containing this table is attached. Differences in clinical characteristics between clusters 0 and 1. The variables are sorted by descending significance. P-values were calculated with a Wilcoxon rank sum test for continuous and ordinal variables and or a Chi-squared test for discrete and binary variables and asses if there are differences in variable distribution between these clusters.

## Abbreviations

*ADAM29*: ADAM metallopeptidase domain 29
BD: Bronchodilator
BODE: Body mass index, airflow obstruction, dyspnea, and exercise capacity
CAT: COPD assessment test
COPD: Chronic obstructive pulmonary disease
COPDGene: Genetic epidemiology of COPD study
CT: Computed tomography
*CTNNA2*: Catenin Alpha 2
DLCO: Diffusing capacity for carbon monoxide
*DSP*: Desmoplakin
eQTL: Expression quantitative trait locus
FEF 25–75: Forced expiratory flow over the middle one half of FVC
FEV_1_: Forced expiratory volume in 1 s
*FGF9*: Fibroblast growth factor 9
FRC: Functional residual capacity
FVC: Forced vital capacity
GOLD: Global initiative for chronic obstructive lung disease
*GSTM1*: Gluthathione S-transferase μ 1
GWAS: Genome-wide association study
HU: Hounsfield units
LAA-950: Low-attenuation area less than -950 Hounsfield units
MESA: Multi-ethnic study of atherosclerosis
MMRC: Modified Medical Research Council Dyspnea Scale
*MRGPRE*: MAS related GPR family member E
*MUC16*: Mucin 16, cell surface associated
PBMC: Peripheral blood mononuclear cell
PCA: Principal component analysis
Perc15: The 15th percentile
PRISm: Preserved ratio impaired spirometry
SGRQ: St. George’s Respiratory Questionnaire
*SLC44A5*: Solute Carrier Family 44 Member 5
ssNPA: Single sample Network Perturbation Assessment
TLC: Total lung capacity
*ZMAT4*: Zinc Finger Matrin-Type 4

## Declarations

### Availability of data and materials

Information about how to access the human cohort data can be found at http://www.copdgene.org and http://www.mesa-nhlbi.org.
