# Supplementary material for: Distinct COPD subtypes in former smokers revealed by gene network perturbation analysis
Source: Respir Res. 2023 Jan 25;24:30. doi: 10.1186/s12931-023-02316-6 (PMC9875487; doi:10.1186/s12931-023-02316-6)
Supplement: Supplementary file 1 — Additional file 1: Figure S1. PCA of COPDGene primary analysis RNA-seq data colored according to batch (A) before and (B) after batch correction. Batch detection with guided principal component analysis showed strong batch effects before batch correction (p < 0.001) that were removed after batch correction (p = 0.538). Figure S2. PCA of COPDGene validation RNA-seq dataset colored according to batch (A) before and (B) after batch correction. Batch detection with guided principal component analysis showed strong batch effects before batch correction (p = 0.001) that were removed after batch correction (p = 0.937). Figure S3. PCA of MESA validation RNA-seq dataset colored according to batch (A) before and (B) after batch correction. Batch detection with guided principal component analysis showed strong batch effects before batch correction (p = 0.003) that were removed after batch correction (p = 1). Figure S4. Clustering tree illustrates the stability of clusters over a range of values for clustering resolution (res). We chose 4 as optimal number of clusters, because cluster number and content (samples) remains constant for res=0.6 to 0.9. When res>0.9 produced some subclusters of these four, but samples did not move across the four branches extending from these clusters. Figure S5. PC elbow plot of the COPDGene discovery set ssNPA features. We heuristically chose 6 principal components for clustering because they captured a large percentage of the variance in the data. Figure S6. Clustering tree illustrates the stability of clusters over a range of values for k in the kNN classification for the COPDGene validation analysis. We chose k=3 because the clusters were stable by this value. Figure S7. Clustering tree illustrates the stability of clusters over a range of values for k in the kNN classification for the MESA validation analysis. We chose k=3 because the clusters were stable by this value. Figure S8. Participant GOLD stage composition according to cluster. The r [file 12931_2023_2316_MOESM1_ESM.docx]

**Online Data Supplement**

**Distinct COPD subtypes in former smokers revealed by gene network perturbation analysis**

**Supplementary Methods**

### Data preprocessing

Several steps were used to process the raw count RNA-seq data prior running ssNPA. First, genes with no reads in any sample were removed and Biomart was used to select only protein coding genes (19,427 in total) (1). RNA-seq counts were transformed to log2 counts per million through mean-variance modeling by the voom function (Limma v. 3.40.6) (2). Because COPDGene is a large study across multiple centers, RNA-seq encompassed 17 batches. We used the batchdetect (gPCA R package, v. 1.0) to detect batch effects with guided principal component analysis (3). Before correction, we observed strong batch effects (p < 0.001), with clear differences among several groups of batches when visualized with the first two principal components of the guided PCA (Supplementary Figure S1A). In order to correct for these effects, we applied the removeBatchEffect (Limma v 3.40.6) (2). After correction, the guided principal component analysis no longer detected any batch effects (p = 0.538, Supplementary Figure S1B). Next, we selected the top 3,000 most variant genes for ssNPA efficiency. Finally, because of the overwhelming effects of smoking on gene expression profile (4, 5), we considered only those participants who were reported as former smokers in both visits. This left us with 617 former smokers with expression measured over 3,000 genes for analysis.

Analogous but independent preprocessing steps were applied to the validation set of 1,444 COPDGene RNA-seq samples, including batch correction (Supplementary Figure S2). However, in this case, the data were filtered to keep the same 3,000 genes selected in the primary analysis. Again, we restricted to participants who were reported as former smokers in both visits, for a total of 763 former smokers for validation. Similarly, the MESA validation set of 821 RNA-seq samples from PBMCs from unrelated individuals at Exam 5 were also preprocessed. Batch correction was performed to remove effects from sequencing site and plate (Supplementary Figure S3) and the same 3,000 genes were retained for subsequent analysis. These data included 431 former smokers for validation analysis.

**COPD subphenotype identification**

We used ssNPA to analyze the training cohort (6). The reference gene expression network was learned using FGES with a penalty discount PD = 11 and cluster resolution res = 0.6. This resolution was chosen after inspection of the cluster stability tree (7) (Supplementary Figure S4). ssNPA trains linear regression models on the reference samples for every gene. The genes in the Markov blanket around the target gene are used as the independent variables in the regression model. ssNPA features in each COPD case sample are the magnitude of the difference between observed and predicted values of each gene (prediction is based on its linear model). Finally, using the COPD samples represented in this new feature space as our dataset, we clustered them in Seurat (8) using the first six principal components of the data (Supplementary Figure S5) and visualized the results with a t-SNE plot (9).

**Assignment of new samples to ssNPA clusters**

RNA-seq samples from the COPDGene validation group were assigned to one of the subphenotypes using the following procedure. For each sample in the validation group, we calculated the network perturbation feature vector as we did when we were analyzing the data from the primary analysis. We used the same reference network to calculate the magnitude of deviations between the validation set reference group and each sample. Then we used the same PCA loadings from the primary analysis to map each new sample to the same PCA space. For the final subphenotype assignment, we used k-nearest neighbor classification with k = 3 (Supplementary Figures S6 and S7).

**Additional Figure S1.** PCA of COPDGene primary analysis RNA-seq data colored according to batch (A) before and (B) after batch correction. Batch detection with guided principal component analysis showed strong batch effects before batch correction (p < 0.001) that were removed after batch correction (p = 0.538).

**Additional Figure S2.** PCA of COPDGene validation RNA-seq dataset colored according to batch (A) before and (B) after batch correction. Batch detection with guided principal component analysis showed strong batch effects before batch correction (p = 0.001) that were removed after batch correction (p = 0.937).

**Additional Figure S3.** PCA of MESA validation RNA-seq dataset colored according to batch (A) before and (B) after batch correction. Batch detection with guided principal component analysis showed strong batch effects before batch correction (p = 0.003) that were removed after batch correction (p = 1).

**Additional Figure S4.** Clustering tree illustrates the stability of clusters over a range of values for clustering resolution (res). We chose 4 as optimal number of clusters, because cluster number and content (samples) remains constant for res=0.6 to 0.9. When res>0.9 produced some subclusters of these four, but samples did not move across the four branches extending from these clusters.

**Additional Figure S5.** PC elbow plot of the COPDGene discovery set ssNPA features. We heuristically chose 6 principal components for clustering because they captured a large percentage of the variance in the data.

**Additional Figure S6.** Clustering tree illustrates the stability of clusters over a range of values for k in the kNN classification for the COPDGene validation analysis. We chose k=3 because the clusters were stable by this value.

**Additional Figure S7.** Clustering tree illustrates the stability of clusters over a range of values for k in the kNN classification for the MESA validation analysis. We chose k=3 because the clusters were stable by this value.


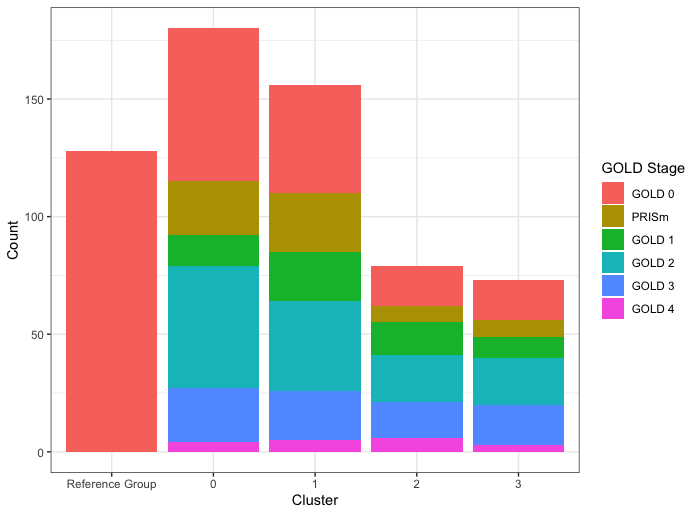


**Additional Figure S8.** Participant GOLD stage composition according to cluster. The reference group was composed of only GOLD 0 participants by design.

**Additional Figure S9.** Clustering based on FEV1 percent predicted does not sufficiently separate COPD individuals with different mortalities..

**Additional Figure S10.** COPDGene validation samples were projected into the same PCA space as the discovery analysis and assigned to clusters with kNN. Density clouds show the distribution of samples in each cluster from the discovery analysis. Individual points represent validation COPDGene samples and are colored according to the cluster to which they were assigned.

**Additional Figure S11.** Heatmaps display the p-value bins for the inter-cohort pairwise comparisons of cluster means by Wilcoxon test for: **physiology** **(A)** FEV1 percent predicted, **(B)** FEV1/FVC, **(C)** DLCO, **(D)** FRC/DLC ratio, **(E)** distance walked in 6 minutes; **symptoms (F)** SGRQ total score, **(G)** MMRC dyspnea score, and **(H)** CAT score. The upper right triangle shows the pairwise comparisons between cluster in the COPDGene discovery set, and the lower left triangle shows the comparisons between clusters in the COPDGene validation set. Blue (red) arrows indicate concordance in the significance (non-significance) of the comparisons between the two cohorts.

*Abbreviations:* FEV1: forced expiratory volume in 1 second; FVC = forced vital capacity; SGRQ = St. George’s Respiratory Questionnaire; MMRC = Modified Medical Research Council Dyspnea Scale; CAT = COPD Assessment Test

**Additional Figure S12.** MESA validation samples were projected into the same PCA space as the discovery analysis and assigned to clusters with kNN. Density clouds show the distribution of samples in each cluster from the discovery analysis. Individual points represent validation MESA samples and are colored according to the cluster to which they were assigned.

**Additional Figure S13.** Heatmaps display the p-value bins for the pairwise comparisons of cluster means by Wilcoxon test for (A) FEV1 percent predicted, (B) FEV1/FVC, (C) FEF 25-75%, and (D) percent emphysema. The upper right triangle shows the pairwise comparisons between cluster in the COPDGene discovery set, and the lower left triangle shows the comparisons between clusters in the COPDGene validation set. Blue (red) arrows indicate concordance in the significance (non-significance) of the comparisons between the two cohorts

*Abbreviations:* FEV1: forced expiratory volume in 1 second; FVC = forced vital capacity; FEF 25-75% = forced expiratory flow over the middle one half of FVC; HU = Hounsfield units

**Additional Figure S14.** ssNPA feature values show a difference in the degree of deregulation of (A) MUC16, (B) ZMAT4, (C), GSTM1, (D) CTNNA2, (E) MRGPRE, (F) SLC44A5, (G) ADARB2, and (H) ADAM29 across clusters. Wilcoxon test p-values highlight where there are differences in the distributions between clusters 0 and 1 and between clusters 2 and 3.

**Additional Table S1.** Excel file containing this table is attached. Clinical characteristics of COPD participants vary across clusters. The variables are sorted by descending significance. P-values were calculated with a Kruskal-Wallis test for continuous and ordinal variables and or a Chi-squared test for discrete and binary variables and asses if there are differences in variable distribution among clusters. Variable means (standard deviations) are also reported for all COPD participants overall, each COPD cluster, and all control subjects for comparison.

BD: bronchodilator; BODE: body mass index, airflow obstruction, dyspnea, and exercise capacity; CAT: COPD Assessment Test; FEF2575: forced expiratory flow over the middle one half of the FVC; FEV1: forced expiratory volume in 1 second; FEV6: forced expiratory volume in 6 seconds; FVC: forced vital capacity; HADS: hospital anxiety and depression scale; MMRC: Modified Medical Research Council Dyspnea Scale; PEF: peak expiratory flow; Perc15: Hounsfield unites below which 15% of voxels lie; Pi10: bronchial wall thickness of inner perimeter of a 10 mm diameter airway; PRM: parametric response map; SF-36: short-form 36-item questionnaire; SGRQ: St. George’s Respiratory Questionnaire

**Additional Table S2.** Excel file containing this table is attached. Differences in clinical characteristics between clusters 0 and 1. The variables are sorted by descending significance. P-values were calculated with a Wilcoxon rank sum test for continuous and ordinal variables and or a Chi-squared test for discrete and binary variables and asses if there are differences in variable distribution between these clusters.

BD: bronchodilator; BODE: body mass index, airflow obstruction, dyspnea, and exercise capacity; CAT: COPD Assessment Test; FEF2575: forced expiratory flow over the middle one half of the FVC; FEV1: forced expiratory volume in 1 second; FEV6: forced expiratory volume in 6 seconds; FVC: forced vital capacity; HADS: hospital anxiety and depression scale; MMRC: Modified Medical Research Council Dyspnea Scale; PEF: peak expiratory flow; Perc15: Hounsfield unites below which 15% of voxels lie; Pi10: bronchial wall thickness of inner perimeter of a 10 mm diameter airway; PRM: parametric response map; SF-36: short-form 36-item questionnaire; SGRQ: St. George’s Respiratory Questionnaire

**Additional Table S3.** Analysis of various COPDGene comorbidities did not show any significant difference between the four identified subtypes. All comorbidities recorded at the time blood samples were collected. *p*-val: chi-square *p*-value.

|  | **All Control Patients** | **All COPD Patients** | **Cluster 0** | **Cluster 1** | **Cluster 2** | **Cluster 3** | **p-val** |
| --- | --- | --- | --- | --- | --- | --- | --- |
| **Number of Subjects** | 128 | 489 | 181 | 156 | 79 | 73 |  |
| High Cholesterol | 62 (48.44) | 254 (51.94) | 90 (49.72) | 83 (53.21) | 47 (59.49) | 34 (46.58) | 0.376 |
| Obese | 56 (43.75) | 187 (38.24) | 72 (39.78) | 60 (38.46) | 30 (37.97) | 25 (34.25) | 0.878 |
| HayFev | 42 (32.81) | 172 (35.17) | 64 (35.36) | 53 (33.97) | 29 (36.71) | 26 (35.62) | 0.979 |
| Stomach Ulcers | 7 (5.47) | 46 (9.41) | 17 (9.39) | 15 (9.62) | 6 (7.59) | 8 (10.96) | 0.915 |
| Sleep Apnea | 17 (13.28) | 107 (21.88) | 38 (20.99) | 38 (24.36) | 17 (21.52) | 14 (19.18) | 0.811 |
| High Blood Pressure | 60 (46.88) | 267 (54.6) | 96 (53.04) | 76 (48.72) | 52 (65.82) | 43 (58.9) | 0.075 |
| CHD | 16 (12.5) | 103 (21.06) | 38 (20.99) | 32 (20.51) | 20 (25.32) | 13 (17.81) | 0.716 |
| GastroEsophReflex | 38 (29.69) | 183 (37.42) | 70 (38.67) | 60 (38.46) | 28 (35.44) | 25 (34.25) | 0.887 |
| PeriphVasculal | 2 (1.56) | 17 (3.48) | 4 (2.21) | 6 (3.85) | 3 (3.8) | 4 (5.48) | 0.609 |
| Stroke TIA | 4 (3.12) | 36 (7.36) | 11 (6.08) | 10 (6.41) | 8 (10.13) | 7 (9.59) | 0.560 |
| Osteo | 9 (7.03) | 87 (17.79) | 29 (16.02) | 21 (13.46) | 20 (25.32) | 17 (23.29) | 0.073 |
| CongestHeartFailure | 0 (0) | 32 (6.54) | 7 (3.87) | 9 (5.77) | 8 (10.13) | 8 (10.96) | 0.100 |

**Additional Table S4.** The genes with the top 5 loadings for each of the first 6 PCs used for clustering the COPD samples in the training COPDGene dataset. Genes are sorted by decreasing contribution to the clustering (sum of the absolute values of the loadings across the first 6 PCs). Loading value is not provided if gene did not rank among the top 5 loadings for a given PC. The sample clustering is driven by differences in the regulation of these genes.

**References**

1. Smedley D, Haider S, Durinck S, Pandini L, Provero P, Allen J, Arnaiz O, Awedh MH, Baldock R, Barbiera G, Bardou P, Beck T, Blake A, Bonierbale M, Brookes AJ, Bucci G, Buetti I, Burge S, Cabau C, Carlson JW, Chelala C, Chrysostomou C, Cittaro D, Collin O, Cordova R, Cutts RJ, Dassi E, Di Genova A, Djari A, Esposito A, Estrella H, Eyras E, Fernandez-Banet J, Forbes S, Free RC, Fujisawa T, Gadaleta E, Garcia-Manteiga JM, Goodstein D, Gray K, Guerra-Assuncao JA, Haggarty B, Han DJ, Han BW, Harris T, Harshbarger J, Hastings RK, Hayes RD, Hoede C, Hu S, Hu ZL, Hutchins L, Kan Z, Kawaji H, Keliet A, Kerhornou A, Kim S, Kinsella R, Klopp C, Kong L, Lawson D, Lazarevic D, Lee JH, Letellier T, Li CY, Lio P, Liu CJ, Luo J, Maass A, Mariette J, Maurel T, Merella S, Mohamed AM, Moreews F, Nabihoudine I, Ndegwa N, Noirot C, Perez-Llamas C, Primig M, Quattrone A, Quesneville H, Rambaldi D, Reecy J, Riba M, Rosanoff S, Saddiq AA, Salas E, Sallou O, Shepherd R, Simon R, Sperling L, Spooner W, Staines DM, Steinbach D, Stone K, Stupka E, Teague JW, Dayem Ullah AZ, Wang J, Ware D, Wong-Erasmus M, Youens-Clark K, Zadissa A, Zhang SJ, Kasprzyk A. The BioMart community portal: an innovative alternative to large, centralized data repositories. *Nucleic Acids Res* 2015; 43: W589-598.

2. Ritchie ME, Phipson B, Wu D, Hu Y, Law CW, Shi W, Smyth GK. limma powers differential expression analyses for RNA-sequencing and microarray studies. *Nucleic Acids Res* 2015; 43: e47.

3. Reese SE, Archer KJ, Therneau TM, Atkinson EJ, Vachon CM, de Andrade M, Kocher JP, Eckel-Passow JE. A new statistic for identifying batch effects in high-throughput genomic data that uses guided principal component analysis. *Bioinformatics* 2013; 29: 2877-2883.

4. Vink JM, Jansen R, Brooks A, Willemsen G, van Grootheest G, de Geus E, Smit JH, Penninx BW, Boomsma DI. Differential gene expression patterns between smokers and non‐smokers: cause or consequence? *Addiction biology* 2017; 22: 550-560.

5. Huan T, Joehanes R, Schurmann C, Schramm K, Pilling LC, Peters MJ, Mägi R, DeMeo D, O'Connor GT, Ferrucci L. A whole-blood transcriptome meta-analysis identifies gene expression signatures of cigarette smoking. *Human molecular genetics* 2016; 25: 4611-4623.

6. Buschur KL, Chikina M, Benos PV. Causal network perturbations for instance-specific analysis of single cell and disease samples. *Bioinformatics* 2020; 36: 2515-2521.

7. Zappia L, Oshlack A. Clustering trees: a visualization for evaluating clusterings at multiple resolutions. *Gigascience* 2018; 7: giy083.

8. Satija R, Farrell JA, Gennert D, Schier AF, Regev A. Spatial reconstruction of single-cell gene expression data. *Nat Biotechnol* 2015; 33: 495-502.

9. Van der Maaten L, Hinton G. Visualizing data using t-SNE. *Journal of machine learning research* 2008; 9.
